# The Ancient Olive Trees (*Olea europaea* L.) of the Maltese Islands: A Rich and Unexplored Patrimony to Enhance Oliviculture

**DOI:** 10.3390/plants12101988

**Published:** 2023-05-15

**Authors:** Valentina Passeri, Clayton Sammut, David Mifsud, Andrea Domesi, Vitale Stanzione, Luciana Baldoni, Soraya Mousavi, Roberto Mariotti, Saverio Pandolfi, Nicola Cinosi, Franco Famiani, Marina Bufacchi

**Affiliations:** 1 Institute for Agricultural and Forest Systems in the Mediterranean, National Research Council, 06128 Perugia, Italy; 2 Institute of Earth Systems, Division of Rural Sciences and Food Systems, University of Malta, 2080 Msida, MSD, Malta; 3 Institute of Biosciences and Bioresources, National Research Council, 06128 Perugia, Italy; 4 Department of Agricultural, Food and Environmental Sciences, University of Perugia, Borgo XX Giugno, 06121 Perugia, Italyfranco.famiani@unipg.it (F.F.)

**Keywords:** ancient olives, health compounds, FAMEs, oil content, phenols, table olives

## Abstract

A prospecting campaign in the Maltese Islands has ensured the survival of several ancient olive trees (*Olea europaea* L.), genetically distant from known cultivars. Most of these plants were abandoned or partially cultivated. A two-year evaluation of fruit characteristics and compositions was performed on samples collected from the main representatives of these indigenous genotypes. Analyses were carried out using Gas Chromatography, High-Performance Liquid Chromatography and Near Infrared Spectrometry. Among the fruit samples, a wide range of variations was observed. Some of the genotypes showed fruit traits suitable for table olive production. This is the case of samples with a pulp/pit ratio higher than four, such as 1Wardija, 1Caritas, 1Plattini, 1Bingemma Malta and 3Loretu, whilst 1Bidni, 1Mellieha, 2Qnotta, 3Loretu, 1Bingemma Malta and 1Caritas were suitable for dual purpose. The total phenol content ranged from 6.3 (1Wardija) to 117.9 (2Mtarfa) g/kg of fresh pulp. The average percentage of MUFA was quite low for most of the varieties. These genotypes, which presumably originated in the Maltese Islands and are well adapted to the local pedo-climatic conditions, are being propagated for the following evaluation of their bio-agronomical performance (production, suitability to intensive cultivation, environmental sustainability, product quality, etc.). The purpose is to select, among these local genotypes, the most outstanding varieties, in terms of phenolic and FA profile and agronomical potential, to spread into cultivation, thereby contributing to an increase in the quality of the local table and olive oil production, strongly linked to the territory.

## 1. Introduction

Extra virgin olive oil is the main ingredient of the Mediterranean diet thanks to its unique nutritional properties. According to the latest estimates of the International Olive Council, Spain, Greece, Portugal, Italy and Cyprus are the European countries with the highest consumption of olive oil, in a range from 11.2 to 6.5 kg of olive oil per inhabitant. However, in 2019 and 2020, among the Mediterranean countries, Malta consumed only 0.2 kg of olive oil per inhabitant [1].

The Maltese archipelago was a center for the cultivation of olive trees and the production of olive oil. Olive pollen was found in samples from the Neolithic period [2] and the Bronze Age [3], even if the introduction of the plant to the archipelago dates back to the Phoenicians in the first millennium BC [4,5]. It was throughout the Roman Empire that the olive industry became very important for the Mediterranean and the Maltese Islands, as evidenced by the numerous remains of ancient mills and oil cisterns. The names of local villages are often associated with olives, such as Ħaż-Żebbuġ (the village of olives), Għajn Żejtuna (oil spring), Birżebbuġa (the well of olives), Iż-Żejtun (olive tree) and Iż-Żebbuġ (olive fruit), confirming that olive trees grew abundantly in these places [6]. Afterwards, olives were replaced with the production of cotton brought by the Arabs, then with other agricultural and pastoral productions, as the inhabitants abandoned agriculture in favor of other, more profitable, activities.

Although the Maltese archipelago has optimal geoclimatic characteristics for olive growing, the whole olive oil sector is struggling to become relevant from an economic point of view. There are several challenges which this industry needs to face, in order to regain interest. Current Maltese agriculture is based on small plots of land owned or cultivated by individual farmers; the unit cost of land is high, as is the cost of labor. Further concern is due to the current climate change scenario, determined by a progressive increase in temperatures and a decrease in rain frequency, which has led to fluctuating oil production in the last few years [7]. Given these constraints, Maltese olive production will hardly be able to reach the high productivity standards of other countries, but it could increase the quality level by exploiting the large indigenous olive genetic heritage that is still unexplored.

The Project for the Revival of the Indigenous Olive (PRIMO) has given a strong impetus to the resumption of olive cultivations even if, in the last two decades, there has been a strong introduction of foreign cultivars, such as the Italian Carolea, Frantoio, Leccino and Pendolino, and table olives including Uovo di Piccione and Bella di Spagna [8]. However, a pool of ancient Maltese olive trees still exists. These include two native Maltese cultivars, Malti and Bidni, and the historically renowned Leucocarpa (with white fruits), Bajda. Alongside these, there are also numerous wild olive trees [9]. Malti is the most widespread variety, due to its high productivity and tolerance to abiotic and biotic stresses. Bidni is believed to be the oldest cultivar, dating back to Roman times [10], although studies based on radiocarbon dating have dated it to the mid–late medieval period, before 1450–1669 AD [11]. The ancient Bidni trees located in the Bidnija area are listed in UNESCO’s Database of National Cultural Heritage Laws, although, since they are living monuments, it is considered more appropriate to speak about “biocultural heritage” [12,13]. Bidni trees produce small fruits which are difficult to harvest, and this has contributed to the progressive abandonment of this cultivar over time. Today, Bidni have been re-evaluated for their good yield of oil, rich in nutraceutical phenolic compounds, especially oleocanthal [14], which gives the pungent flavor and spicy sensation. Bajda trees produce white ripe olives, and the Knights of Order of Saint John probably introduced them to Malta. These olives were rediscovered in 2010, thanks to the presence of a few trees in a garden belonging to the Knights, and today are locally appreciated as a table olive due to their sweet pulp [15].

Olive cultivars may be distinguishable based on their morphological characteristics [16,17], but some traits are strongly influenced by the agro-environmental conditions [18]. A big step forward for the identification of olive cultivars has been made by the application of molecular markers [19,20,21,22]. Mazzitelli and colleagues [9], by applying molecular analysis to the local olives of the islands, highlighted a clear separation between the two main cultivars: Bidni and Malti, with the latter being related to the San Anton variety, a giant tree considered a wild-type oleaster. di Rienzo and colleagues [23], using SSR markers on the varieties of four countries (Italy, Algeria, Syria and Malta), showed that Italian and Maltese populations both appeared to derive from the Syrian one, probably due to the migration routes of the Phoenicians [23]. In addition, in a recent and in-depth work [24], it has been highlighted how rich in olive diversity the small islands of Malta still are, representing a center of hybridization with a selection of local olive genotypes, with some samples possibly claiming an autochthonous origin. The combination of nuclear and plastidial markers of a large sampling set of centennial olives spread over the Maltese Islands showed that several trees were grafted, confirming that this technique has been known since ancient times [9,23].

In the present study, the evaluation of several aspects concerning the characteristics of the fruits, the acidic, phenolic, squalene and sterol compositions of the most representative native olive genotypes of Malta, was performed for the first time. Many of these genotypes were long abandoned and, in some cases, difficult to reach and with limited production, mainly because of the few resources available to the olive trees. For this reason, and to eliminate any interference caused by the olive oil extraction process, the fruits were directly analyzed, leaving the oil analysis for a future step of the work. In any case, most of the chemical compounds studied in olive oil or their direct precursors are already present in the pulp of the olive fruits, generally at higher concentrations. Therefore, the direct evaluation of the metabolites contained in the drupes may give direct indications of the potential of a genotype for the production of high value oil [25], without the interference of the transformation and conservation processes. These native genotypes, representing the great olive diversity existing in Malta, once characterized, could offer a major contribution to an increase in the quality of local extra virgin olive oil and table olives. Furthermore, the local genotypes could reveal interesting bio-agronomic characteristics, making them a resource to be fully exploited [26] in order to face new production needs and climate change.

## 2. Results

### 2.1. Fruits Harvested from Indigenous Maltese Genotypes Had Traits Suitable for Oil Production and for the Preparation of Table Olives

**Figure 1:**
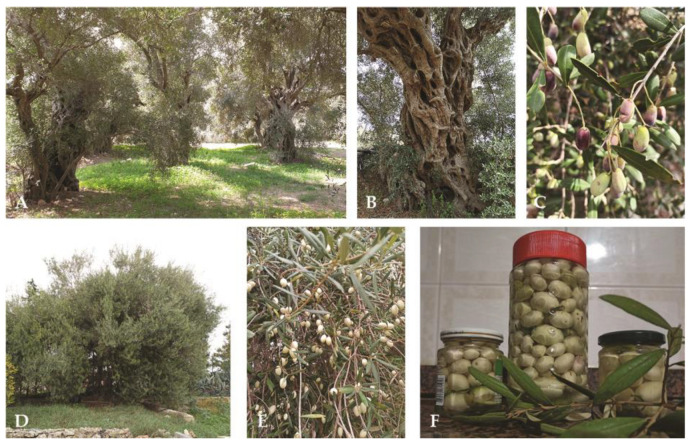
Ancient trees and their fruits from Bidni (**A**–**C**) and Bajda genotypes ((**D**,**E**)—in this paper it is most likely assumed to be 1Caritas sample) with home-prepared white olives, brined with rosemary and lemon, according to tradition (**F**).

Fruits of 19 genotypes grown on Maltese territory were collected and evaluated over the course of the 2020 and 2022 crop seasons. Among these, two were traced back to Italian cultivars: Nocellara del Belice (1Plattini) and Ottobratica (3Gudja), while the other 17 represented potentially native Maltese plants [24] including Bidni (1Bidni, Figure 1A–C) and the Leucocarpa 1Caritas plant (Figure 1D–F).

**Figure 2:**
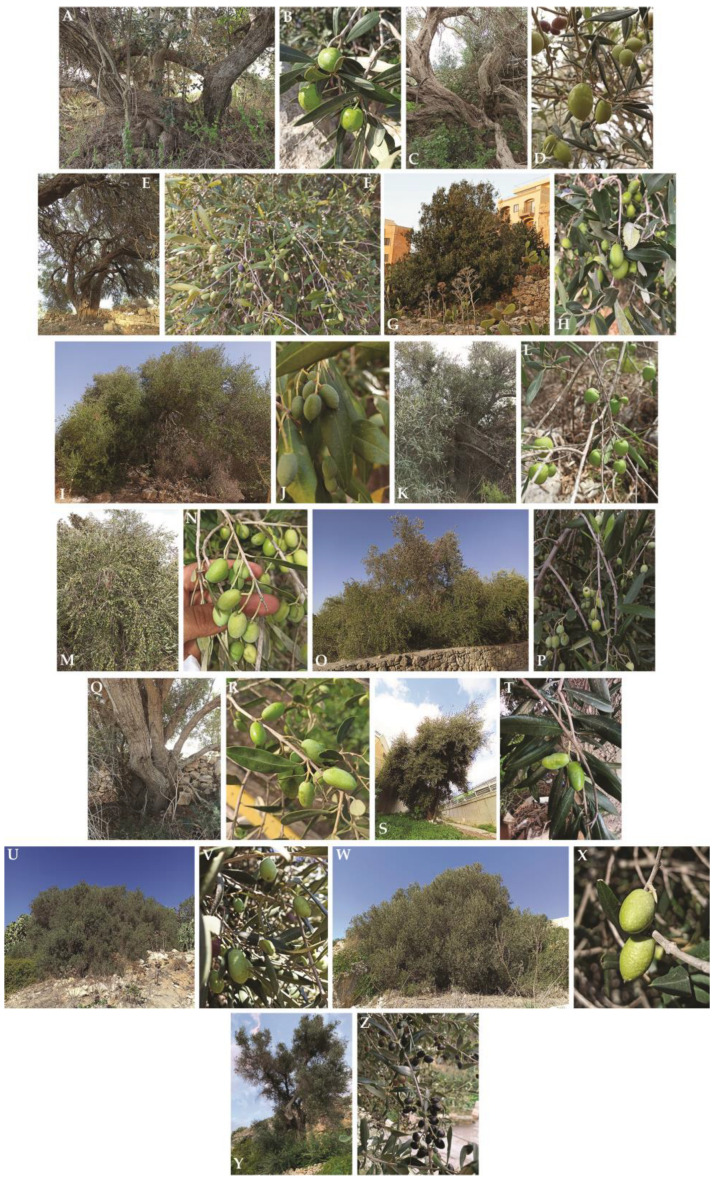
Some olive trees and their sampled drupes. 1Plattini (**A**,**B**); 1Bingemma Malta (**C**,**D**); 1Haz Zebbug (**E**,**F**); 1Mellieha (**G**,**H**); 1Pembroke (**I**,**J**); 1Wardija (**K**,**L**); 2Wardija (**M**,**N**); 2Gudja (**O**,**P**); 3Gudja (**Q**,**R**); 2Kappara (**S**,**T**); 5Mtarfa (**U**,**V**); 6Mtarfa (**W**,**X**); 1Lunzjata (**Y**,**Z**).

Most of the plants were abandoned, near slopes, roadsides, uncultivated lands or in private gardens (Figure 2 and Table 1), and it was not always possible to find fruit in the quantity and time required.

A wide range of variations in the characteristics of the examined fruits was observed between the samples and in the two seasons (Table 2) under investigation. 1Wardija, 1Plattini and 1Bingemma Malta had the heaviest weight (fresh fruit weight, FW), with values ranging between about 7 g and more than 3 g, on average in the two years. Even 1Caritas had a weight of 5.77 g in 2022, against a weight of just over 3 g in 2020, when the fruits were harvested in mid-September. The fruits of 1Mellieha, 3Gudja, 2Qnotta, 3Loretu, 2Kappara and 2Wardja had an FW between 2.58 g and 1.29 g, while in the remaining samples the fruits weighed under 1 g, with the minimum value of 0.40 g in 2Pembroke and 6Mtarfa in 2022.

1Wardija, 1Caritas, 1Plattini, 1Bingemma Malta and 3Loretu showed a pulp/pit (P/P) ratio greater than four, meaning they were considered to be suitable for the production of table olives. Some plants produced fruits with a low P/P ratio. This was the case for 1Haz Zebbug, 1Pembroke, 2Mtarfa and 2Pembroke, which were characterized by a very thin pulp and, considering the small size of the fruits, could suggest they were wild or wild-related olive trees. Moreover, 2Mtarfa had pits that often shattered under the slightest manual pressure.

The fruits with a high-water content (fruit moisture, FrM) were 1Lunzjata (64.55%), 1Plattini (64.25%), 2Qnotta (62.46%) and 3loretu (62.44%), while 1Pembroke and 1Haz Zebugg showed the lowest amount of moisture (48.62%).

The drupes analyzed had medium–low oil contents over the two years (Table 2). 1Wardija was the genotype with the highest oil content in the fresh fruit (24.59 and 19%) and in dry weight (58.19 and 45.85%), while 2Mtarfa, 2Kappara, 2Wardija, 1Haz Zebbug, 2Gudja, 1Pembroke and 2Pemboke had values that did not even reach 10% OCFW.

The lowest value was detected in 1Lunzjata with 2.19% oil content in the fresh fruit (Table 2), in 2022. The oil content of the drupes depends on various factors related to the soil and climatic conditions of the growing area, the agronomic management and the degree of ripeness of the olives themselves [27,28,29].

The fresh weight, pulp/pit ratio and the oil content in reference to the fresh weight were used to study the relationships among them, in different combinations (Figure S1). A linear relationship was found between the fresh weight and the pulp/pit ratio indicating that it was the contribution of the pulp to the determination of the weight of the fruit itself (R^2^ = 0.89). Furthermore, as the pulp increased, an increase in the percentage of oil was obtained (R^2^ = 0.82).

### 2.2. The Main Phenolic Compounds Contained in the Maltese Olive Pulp Were Highly Variable

**Figure 3:**
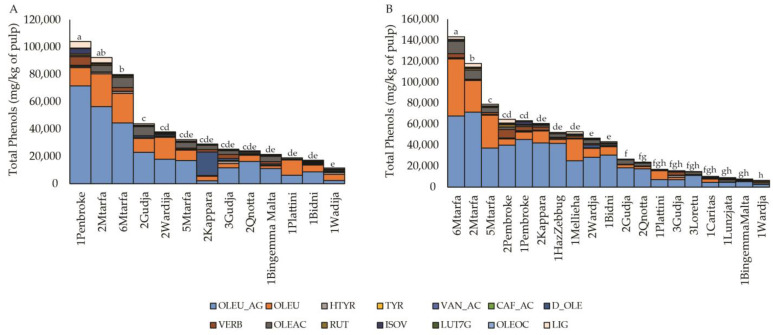
The variation in phenol content in the Maltese olive samples (expressed as mg/kg of pulp fresh fruit) in 2020 (**A**) and 2022 (**B**). Different letters correspond to significantly different values of total phenols, at *p* < 0.05. OLEU_AG: oleuropein-aglycone; OLEU: oleuropein; HTYR: Hydroxytyrosol; TYR: tyrosol; VAN_AC: vanillic acid; CAF_AC: caffeic acid; D_OLE: dimethyl-oleuropein; VERB: verbascoside; OLEAC: oleacein; RUT: rutin; ISOV: isoverbascoside; LUT7G: lutein 7-glycoside; OLEOC: oleocanthal; LIG: ligstroside.

Analyses of all the phenolic compounds in two crop seasons among the samples showed important variations in phenol content (Figure 3 and Table S1). The highest total phenol contents were detected in 6Mtarfa, 2Mtarfa, 1Pembroke and 5Mtarfa with about 143.2, 117.9, 104.1 and 92.3 g/kg of pulp, respectively. Most of the fruits had a total phenol content between 79.9 and 16.2 g/kg regardless of the harvest season, while the lowest values were in 3Loretu, 1Caritas, 1Lunzjata, 1Bingemma Malta and 1Wardija, which ranged from 14.9 to 6.3 g/kg, in 2022 (Table S1).

The main phenolic compounds in all the samples belong to secoiridoids. Among them, the highest value was for the oleuropein aglycone (OLEU_AG), followed by oleuropein (OLEU) and oleacein (OLEAC), with some differences in the quantities for the two years. 2Kappara fruits harvested in 2020 were an exception, as these were particularly rich in dimethyl-oleuropein (D_OLE, 17.2 g/kg of pulp). 1Pembroke, 2Pembroke, 3Gudja and 6Mtarfa were the fruits with the highest concentrations of verbascoside (VERB), while in the other samples, a high variation in the two years was observed. Furthermore, 1Pembroke and 1Lunzjata were the only samples where isoverbascoside (ISOVERB) was detected.

Among the phenol compounds with the greatest variation, both between samples and between seasons, were the phenolic alcohols with hydroxytyrosol (HTYR) and tyrosol (TYR). In all fruits, the contribution of flavones, rutin (RUT) and luteolin-7-glycoside (LUT7G) was important, while lignans were not detected.

### 2.3. Variation of Squalene and Sterol Content in Drupes

Squalene analysis in all 19 genotypes showed a significant difference between them and crop seasons. The highest concentrations were found in 1Plattini, 1Bingemma Malta, 1Wardija, 1Caritas, 2Qnotta and 3Loretu in a range from a maximum of 9.6 g/kg of pulp fresh weight to a minimum of 2.6 g/kg (Table 3). The lowest squalene contents were found in 1Lunzijata, 1Pembroke and 2Pembroke and 1Haz-Zebbug, with a range between 33 and 14 mg/kg of pulp (Table 3).

Analyses of sterols showed, as with the previous bioactive compound, considerable variation. Very interestingly, the sterol composition in all 19 Maltese samples was represented exclusively by β-sitosterol, being the only compound detected by the instrumentation, under the analysis conditions used. The plants with the highest content of total sterols were 2Kappara, 1Bingemma Malta, 1Bidni and 1Wardija with values ranging from about 550 to 405 mg/kg, while those with the lowest content were 2Mtarfa and 1Pembroke with values ranging from 98 to 61 mg/kg (Table 3), in 2020. The highest variation in sterol content between the two harvest seasons was measured in 1Bidni, 1Bingemma Malta, 3Gudja, 2Kappara, 1Plattini and 1Wardija, and the least variation was measured in 5Mtarfa, 2Qnotta and 2Wardija (Table 3).

### 2.4. The Acidic Composition in the Fruit Pulp of 19 Maltese Olive Trees

The content of fatty acids in the drupe pulp showed a high variation both between the samples and in the two years. The oleic acid (C18:1) content reached the maximum value equal to 70.48% in 1Bingemma Malta, while the minimum was found in 2Pembroke with 32.49%, in 2022 (Table S2). Moreover, the fruits of 1Wardija, 2Kappara and 2Mtarfa contained oleic acid below 55% in both years, while 1Pembroke (45.59%), 1Plattini (49.66%), 2Wardija (46.02%) and 2Pembroke showed values lower than 50% (Table S2).

Linoleic acid (C18:2) showed a fair variability between the analyzed fruits and in the years analyzed, even reaching values close to 20%. The maximum value of linoleic acid was reached in 1Wardija with 19.92%, while the lowest value was in 2Mtarfa with 6.25% (Table S2).

Particularly high percentages of linolenic acid (C18:3) were found in the following different samples: 1Bingemma Malta, 1Pembroke, 1Wardija, 2Mtarfa, 6Mtarfa, 1Lunzjata and 2Pembroke, with values that greatly exceeded 3% and even reached 7% (in 2Pembroke fruits), in at least one year investigated (Table S2). The highest values of palmitic acid (C16:0) were in 1Wardija, 1Lunzjata and 3Gudja with values from 22.47% to 20.51%, while the lowest value was in 2Mtarfa with 13.28% (Table S2). The highest OLP (oleic acid/linoleic acid + palmitic acid) ratio was found in 3Loretu with a value of 2.89, while the lowest amount was observed in 2Pembroke with a ratio of 1.20 (Table S3). In addition to the main fatty acids just described, the fruits from the 19 Maltese genotypes contain other types of both saturated and unsaturated fatty acids, sometimes in small traces and often below instrumental detection limit. 1Plattini and 1Caritas contained the highest concentration of stearic acid (C18:0), ranging from 5.86% to 5.14% (Table S2), respectively. In all the other samples, the stearic acid ranged between 2.97% and 1.23%, with the 1Bidni genotype displaying the most stable value with 2.27% in both years.

In addition to oleic acid, the monounsaturated fraction (MUFA, Table S3) had the contribution of palmitoleic acid (C16:1), with a range of value from 5.05% (1Lunzjata) to 0.57% (2Wardija), and the contribution of cis-10-heptadecenoic acid (C17:1), with the highest value of 7.51% in 2Kappara and the lowest value in 3Gudja with 0.28% (Table S2). Moreover, in some samples this fraction included the eicosenoic acid (C20:1n9) in 2022 (Table S2). The two forms of eicosatrienoic acid (C20:3n6 and C20:3n3) contributed to the polyunsaturated fraction (PUFA, Table S3) and some samples also contained arachidonic acid (C20:4n6) in rather large percentages (Table S2). The fraction of saturated fatty acids (SFA, Table S3), in addition to palmitic acid (C16:0), included stearic (C18:0), arachidic (C20:0) and henicosanoic (C21:0) acids.

## 3. Discussion

Despite their limited size, low number of habitats and intense human pressure, the Maltese Islands represent an invaluable source of biodiversity in terms of terrestrial and aquatic flora and fauna [30]. The most characteristic terrestrial agro-ecosystems are those represented by the typical species of the Mediterranean, among which the olive tree stands out. Contrary to what was believed until a few years ago [9,23], several potentially indigenous olive genotypes of Malta have recently been brought to light [24]. Evaluations of the fruit characteristics and the main chemical constituents of the fruits harvested from some of these unknown olive trees suggest their possible use in the production of oils and/or olive processing products, strongly linked to the Maltese territory.

The phylogenetic analysis of Maltese genotypes and numerous cultivars from the Mediterranean and beyond have highlighted the presence of distinct groups of individuals spread throughout the Maltese territory [24]. Many of these were not strictly related to known cultivars and therefore can be considered potentially indigenous to the Maltese archipelago.

Wide ranges of variations for some traits were observed among the fruit samples. Those varieties with a high fruit weight, a high pulp/stone ratio and a medium or medium–low oil content, such as 1Wardija, 1Bingemma Malta, 1Caritas and 1Plattini, were deemed suitable for the production of table olives. In particular, in both considered years, 1Wardija showed a fruit size and a pulp/pit ratio similar to those of 1Plattini. The latter corresponded genetically to the Sicilian Nocellara del Belice cultivar, one of the best Italian table olive cultivars, and our results for some of the studied fruit traits are in line with what was reported in previous works [31,32,33]. Moreover, 3 Gudja, which corresponds to the Ottobratica cultivar, had a fruit weight and pulp/pit ratio comparable or slightly higher than that reported in a previous work conducted on the same Italian cultivar, in the Calabria region [16]. The differences found in the two years between some traits of the fruits and the genotypes were probably the result of different amounts of fruit on the trees, and therefore different sizes of the fruits themselves. Unfortunately, due to the difficult location and size of the trees, it was not possible to evaluate the productivity of each year for the various genotypes. In order to establish their product destination, further investigations should be carried out on fruit characteristics, such as fruit and stone shape and size, pulp firmness and ripening index [34,35,36]. 1Mellieha, 3Gudja, 2Qnotta, 3Loretu, 2Kappara and 2Wardija, showing intermediate fruit sizes and medium/medium–low oil contents, should be considered as suitable as double purpose varieties, as well as 1Bidni and 5Mtarfa, which have small fruits and medium/medium–low oil contents. 2Mtarfa, 1Pembroke, 2Pembroke and 1Haz Zebbug, with very small fruits and very low pup/pit ratio and oil contents, have characteristics that could relate to wild oleasters, which are characterized by small fruits, very thin pulp and low oil content. The correlation between small fruit, low oil content and thin flesh in wild olive genotypes is well established. In fact, olives produced from wild olives usually have an oil content between 5 and 10% [37,38,39]. In this study, some genotypes, such as 2Mtarfa and 1Pembroke (both E2), showed very low oil values, at just over 2% of their fresh fruit weights, also as a consequence of the prevalence of the stone with respect to the pulp. Moreover, three out of these four genotypes belong to lineage E2 [24], typical of the wild olives of the central Mediterranean [40]. Conversely, the large and medium sized fruits were traced back to the lineage E1, with the exception of 2Qnotta and 1Bingemma Malta which belonged to lineage E2 [24], as observed in other Mediterranean cultivars [41].

Phenolic compounds were highly variable among all the fruit samples studied in both years. Considering the quantities and the different phenol fractions found, it can be stated that some Maltese genotypes have important radical scavenging, antioxidant and anti-inflammatory properties [42,43,44,45,46]. The main phenolic compounds in the samples under investigation were secoiridoids and their derivatives, as has already been found in other studies [7,8,9,10,11,12,13,14,15,16,17,18,19,20,21,22,23,24,25,26,27,28,29,30,31,32,33,34,35,36,37,38,39,40,41,42,43,44,45,46,47,48,49,50]. The contribution of phenolic alcohols, such as hydroxytyrosol and tyrosol, of flavonoids with rutin and luteolin 7-glycoside and of acid phenols (particularly vanillic acid) was relevant. These compounds are indeed fundamental both for their beneficial effects on human health and for their contribution to the extra virgin olive oil (EVOO) taste giving them pungency and bitterness [51]. It will therefore be appropriate to direct the olive oil production chain to maintain the maximum levels of phenolic compounds [52,53]. The Ottobratica cultivar is known to produce an excellent EVOO from the point of view of oxidative stability thanks to its content in phenols and tocopherols, in Southern Italy conditions [54]. The corresponding Maltese sample, 3Gudja, has a medium–low content of total phenols in the fruit pulp if compared with the other Maltese genotypes investigated. Moreover, the fruits of 1Bidni, 1Mellieha, 1Bingemma Malta, 2Kappara, 2Qnotta and 2Wardija, in at least one year, showed contents of total phenols higher or comparable to those found in the fruit pulp belonging to olive cultivars such as Arbequina, Coratina, Koroneiki, Leccino, Picholine Marocaine and Moraiolo [55]. This suggests that, if processed appropriately, high-quality EVOOs could be obtained from some of the genotypes covered by this research, which also show better parameters than those obtained from well-known olive cultivars. Interestingly, the genotypes with the highest total phenol content corresponded to those related to wild olive trees, such as 2Mtarfa, 5Mtarfa, 6Mtarfa, 1Pembroke and 2Pembroke [24], even though their scarce oil content does not suggest the need to further evaluate them in the perspective of their reintroduction into cultivation. Several research groups [56,57,58,59] have shown that, from wild olive trees, it is possible to obtain oils with a higher content of polyphenols, sterols and fatty acids than oils from cultivated olive trees. In any case, even if these parameters are also affected by environmental and cultural conditions, we can consider these genotypes a source of variability to be preserved and studied to understand their positive features (i.e., resistance to biotic/abiotic stress) and to be used in genetic improvement programs [60]. The genotypes with large fruits and a good percentage of pulp, such as 1Wardija, 3Loretu, 1Bingemma Malta and 1Caritas, had a lower total phenol content per kg of pulp in both crop years. However, this phenol content was still enough to ensure optimal conservation and nutraceutical properties in table olives. The different treatments used to produce table olives are fundamental in reducing the bitterness of the oleuropein [61], but the phenol content remains higher than in EVOO. It is estimated that table olives can contain from 100 to 400 mg/100 g of fruit pulp of simple and complex phenolic compounds, equal to those contained in 1 kg of EVOO [53]. The fruits analyzed for their use as table olives included 1Plattini, corresponding to the Nocellara del Belice cultivar, and the 1Caritas plant. The suitability of 1Caritas to be used for table olives is supported by previous observations/studies in the Leucocarpa olive trees, which show large egg-like white fruits. These white drupes are the result of the destabilization of the production process of various secondary metabolites during the fruits’ development and maturation [62]. Moreover, these white fruits have been reported as sweet and good for being eaten in brine or used as ingredients in traditional Maltese cuisine [15].

1Plattini, 1Wardija, 1Bingemma Malta and 1Caritas had the highest squalene content, ranging from 9.6 to 4.6 g/kg of pulp, in at least one year. These values are much higher than those reported in other works [55,63], but the difference could be, in some cases, due to the genotype, the growth environment or the time of ripening of the fruit. Moreover, 2Mtarfa, 6Mtarfa, 1Pembroke, 2Pembroke, 1Lunzjata and 1Haz Zebbug showed very low concentrations of squalene. These squalene values confirmed what was reported in previous studies in olive fruits [55,63] on cultivars whose fruits were harvested early. Squalene is a lipophilic triterpene and it is a metabolic intermediate of the sterol biosynthesis which plays a role as a scavenger of free radicals and, therefore, in the prevention of various disorders related to the cardiovascular system and as an anticancer agent [64,65,66,67].

The fruits of the genotypes under study contained β-sitosterol as the only main detectable sterol. By sampling the fruits in subsequent periods, we expect a decrease in the value of β-sitosterol and an increase in other forms of sterols, as has already been found in other studies [68]. The sterol fraction of olive oil is receiving increasing attention for its beneficial effects on human health, for detecting adulteration and for verifying the authenticity of oils [69,70,71]. It is therefore essential to take into consideration the high content of sterol compounds when selecting new olive cultivars. To this end, even the Maltese genotypes whose drupe pulp was particularly rich in sterols can represent a resource to be used in genetic improvement programs [70]. The total sterol content in the fruit pulp of the various genotypes under study was highly variable. This was in agreement with what was found in the literature on oils as well [72,73]. Sterols are minor components contained in the unsaponifiable fraction of the oil and, for this reason, the low contents of these compounds could be due to the low oil contents of the fruits. In fact, in our data, total sterols showed a high positive correlation with oil content, both reported as fresh (*r* = 0.751, *p* < 0.01) and dry (*r* = 0.790, *p* < 0.01) fruit weight.

Traditionally, the healthy properties of EVOOs have been attributed to its balanced composition between unsaturated and saturated fatty acids [74]. A fundamental role is played by the high content of MUFA, in particular oleic acid, which represents up to 80% of its total lipid composition [74,75]. The presence of only one double bond in the MUFA molecule makes EVOO more resistant to oxidation, thus contributing to its antioxidant properties, high stability and long life, compared with oils rich in PUFA [74,76,77,78,79]. In the Maltese fruits analyzed, an unbalanced fatty acid composition was found in rS1espect to IOC standards [80]. The oleic acid content (C18:1) was less than 55% in many samples, reaching as low as 32% in 2Pembroke. However, without considering the cases where fruit traits and the oil content were not interesting, the samples of 1Bidni, 2Qnotta and 3Gudja showed an oleic acid content between 56% and 67% in both years. On the contrary, in the fruits of 1Bingemma Malta, 1Mellieha, 3Loretu, 2Gudja, 1Caritas, 1Pembroke and 1Lunzjata, the oleic acid content was between 70% and 58%, in at least one year. Among all these samples however, 3Gudja and 1Lunzijata exceeded the permissible limit (20%) of palmitic acid [80]. Moreover, rather high contents of other saturated fatty acids (C22:0 and C21:0), normally present in traces in EVOOs, were found in several Maltese genotypes [80,81,82]. In all the fruits analyzed, the linoleic acid composition did not exceed the maximum quality limit of 21%; however, linolenic acid was higher than 1%, reaching values of more than 4% in 1Bingemma Malta, 1Pembroke, 1Wardija, 2Mellieha, 6Mtarfa, 2Mtarfa and 1Lunzjata, and up to exceeding 7% in 2Pembroke. These values could be related to what has already observed in case studies in Western Sicily where linolenic acid values of 8.45% were found [54].

Despite having a strong genetic component, the composition of fatty acids is heavily influenced by the crop year and environmental factors which determine the variation in the quality parameters [82,83]. In general, our results show a low oleic acid content and a high palmitic acid content in line with results obtained in similar conditions of the high temperature typical of warm climates. The main data (minimum and maximum temperatures and precipitation) derivate from two different datasets, as reported in Figure S2 and Table S4. Furthermore, the increases in temperature that have occurred in recent years due to climate change tend to create an imbalance in the ratio of MUFA, PUFA and SFA. In particular, the reduction of oleic acid has been related to the increase in temperature in various studies [27,84,85,86,87]. Since the fraction of minor compounds in an oil is strongly conditioned by the type of extraction technology used, the lipid fraction tends to undergo less alteration. For this reason, and on the basis of the combination of fruit traits, oil content and chemical compounds in the pulp, we propose 1Bidni, 2Qnotta, 1Bingemma Malta, 1Caritas, 1Mellieha and 3Loretu as Maltese genotypes to be used for the production of EVOO, due to them having a good composition of fatty acids and minor bioactive compounds.

## 4. Materials and Methods

### 4.1. Sample Collection and Assessment of Drupe Characteristics

The phylogenetic analyses of centuries-old olive trees present in the Maltese territory and numerous cultivars from the Mediterranean and beyond have highlighted the presence of distinct groups of individuals spread throughout the Maltese territory [24]. Many of these were not strictly related to known olive cultivars and therefore could be considered potentially indigenous to the Maltese archipelago. A set of seventeen of these genotypes were used in this study. Two Italian cultivars, Nocellara del Belice and Ottobratica, [24] were chosen as reference plants. Drupes were collected from the plants listed in Table 1 over two years: 2020 and 2022. Furthermore, six samples from 2020 were not comparable with the rest of the fruits and therefore it was decided not to report them in the results. The fruits were randomly chosen around the canopy, collected in nets, carefully packed to avoid damage during transport, shipped quickly and subsequently stored at −80°C until analysis. From each sample, three subsets of about 10 representative fruits were selected to measure fresh fruit weight (FW), fruit moisture (FrM) by drying the drupes in a forced-air oven at 105 °C until constant weight, pulp/pit ratio (P/P) as reported in [87] and the oil content using a Near Infrared Spectrometer (NIR) [88].

The biochemical profiles of the fruits were evaluated using the fresh epicarp and mesocarp, hereinafter referred to as pulp.

### 4.2. Extraction and Analysis of Fruit Phenolic Compounds

The phenolic compounds were extracted from the olive pulp according to previously developed protocols [89,90] with minor modifications. One gram of pulp from several drupes was extracted in 6 mL of DMSO and kept at 4 °C overnight. The extracts were filtered through a 0.45 μm nylon mesh. Syringic acid (Merck, Darmstadt, Germany) was added to each sample to be analyzed as an internal standard at a final concentration of 100 mg/L. The HPLC analyses of the phenolic extracts were conducted by a reversed-phase column using a HPLC-DAD, Agilent 1100 Series, Diode Array Detector [91], using a 2.6 µm Kinetex^®^ Phenyl-Hexyl 100 Å, 100 × 4.6 mm LC Column (Phenomenex^®^, Torrance, CA, USA). Three external standard mixes by single net standards (Merck, Darmstadt, Germany) were prepared to identify the main phenols contained in olives. The quantification of each identified component has been carried out using the internal standard method.

### 4.3. Sterol and Squalene Analyses

The extraction of sterols and squalene from 400 mg of pulp was performed starting from [63] protocol, by alkaline hydrolysis with 2 mL of KOH 2%, in a water bath at 80 °C for 15 min and using 5α-cholestan-3β-ol (Merck, Darmstadt, Germany) as internal standard. The unsaponifiable fraction was extracted with 1 mL hexane and 1.5 mL NaCl 1 %. The upper hexane layer was analyzed by GC-FID (Agilent Technologies 7820A, Santa Clara, CA, USA), using a ZB-5HT Inferno™ capillary column 30 m × 0.25 mm × 0.10 μm film thickness (Phenomenex). The quantification of each identified component has been carried out using the internal standard method.

### 4.4. Fatty Acid Methyl Esters (FAMEs) Analyses

FAs’ composition was analyzed by FA methylation of fruit pulp [92] with minor changes. Then, 300 mg of pulp were extracted with 4 mL of methylation solution (Methanol:Toluene:2,2-Dimethoxypropane:Sulfuric acid in 39:20:5:2 proportion) and 2 mL of heptane. Tubes were transferred in a dry bath for two hours to 80 °C and then let to cool down to room temperature. Then, 1.5 mL of supernatant were analyzed by GC-FID (Varian CP-3800) using a ZB-WAX capillary column 60 m × 0.25 mm × 0.25 μm film thickness (Phenomenex). The Supelco™ 37 Component FAMES Mix (Merck, Darmstadt, Germany) was used to identify key fatty acid methyl esters.

### 4.5. Statistical Analysis

All data were analyzed using One-way ANOVA followed by Tukey’s multiple comparison test [93] of SPSS Statistics version 23 (IBM Corp., Armonk, NY, USA). All analyses were performed at a significance level of *p* < 0.05. The linear regression between some parameters of the fruit and the oil content was evaluated using the same software. The correlations between oil content, both fresh and dry weights and total sterols were analyzed using Pearson’s correlations. All analyses were carried out for three technical replicates of each fruit sample in two different crop seasons.

## 5. Conclusions

The fruits of seventeen potentially autochthonous olive genotypes of the Maltese Islands were characterized by their fruit traits and main biochemical compounds. Some of these genotypes had better characteristics when compared with known Italian and Maltese olive cultivars, growing under comparable climatic conditions. In particular, 1Wardija, 1Bingemma Malta and 1Caritas have shown a good aptitude to be used as table olives compared with 1Plattini (Nocellara del Belice), thanks to the combination of large fruits, with optimal pulp/pit ratios and satisfactory contents of bioactive compounds (Table S5). Other genotypes were more suitable for the production of EVOO, considering their oil content and good levels of the main fatty acids, in particular their oleic acid and nutraceutical component profiles. Among these, we can mention 1Bidni, 1Mellieha, 2Qnotta, 3Loretu, 1Bingemma Malta and 1Caritas, which can be considered suitable for dual-purpose use (table and olive oil, Table S5). Furthermore, some genotypes, although showing particularly high values for the main bioactive compounds, are not suitable for production purposes due to their small fruit size. This is the case for 1Haz Zebbug, 1Pembroke, 2Mtarfa and 2Pembroke, which are characterized by a very thin pulp and low oil content. However, they must be preserved and further studied as they may represent a unique source of genetic variability to be exploited in genetic improvement programmes.

These genotypes are being propagated and will be cultivated in the best possible conditions to fully study their bio-agronomic features during several years of observations and in uniform environmental and agronomic conditions. Furthermore, to obtain more indications on the optimum harvesting time and on maximizing the quality of the productions for each genotype, it is necessary to evaluate the ripening trend of both the characteristics of the fruit and the biochemical composition.

An in-depth study of the agronomic characteristics and adaptations to extreme conditions can highlight useful genotypes to be exploited to overcome the new demands of production, cultivation and climate change.

A novel Maltese oliviculture and olive oil production strongly linked to the Maltese territory can originate in the near future from the use of some of these ancient olive plants.

## Figures and Tables

**Table 1:** List of Maltese olive samples collected. The table was elaborated from [24]. Information on the location of the plants can be found in the same cited article.

Sample Name	Matched Genotype	Cultivation Area and Tree Status, Brief Description
1Bidni	Bidni	Deep soil in a cultivated field. Status of tree: not abandoned but needs to be taken care of
1Plattini	Nocellara del Belice	Deep soil in an uncultivated field. Status of tree: abandoned
1Bingemma Malta	Unknown	Deep soil in a cultivated field. Status of tree: abandoned
1Caritas	Leucocarpa	Deep soil in a cultivated orchard. Status of tree: good maintenance
1Haz Zebbug	Unknown	Deep soil in an uncultivated field. Status of tree: abandoned
1Mellieha	Unknown	Shallow rocky soil in a garigue habitat in an uncultivated field. Status of tree: abandoned
1Pembroke	Unknown	Medium level of soil in a maquis habitat in a valley. Status of tree: abandoned
2Pembroke	Unknown	Medium level of soil in a maquis habitat in a valley. Status of tree: abandoned
1Wardija	Unknown	Deep soil slope in a maquis/woodland environment. Status of tree: abandoned
2Wardija	Unknown	Deep soil slope in a maquis/woodland environment. Status of tree: abandoned
2Qnotta	Unknown	Deep soil in a cultivated field. Status of tree: abandoned
2Gudja	Unknown	Deep soil in an uncultivated field. Status of tree: abandoned
3Gudja	Ottobratica	Deep soil in a cultivated field. Status of tree: abandoned
2Kappara	Unknown	Deep soil in an uncultivated patch of the field. Status of tree: abandoned
2Mtarfa	Unknown	Rocky shallow soil in a transition between a garigue and maquis environment. Status of tree: abandoned
5Mtarfa	Unknown	Rocky shallow soil in a transition between a garigue and maquis environment. Status of tree: abandoned
6Mtarfa	Unknown	Rocky shallow soil in a transition between a garigue and maquis environment. Status of tree: abandoned
3Loretu	Unknown	Deep soil in a cultivated garden. Status of tree: fair maintenance
1Lunzjata	Unknown	Deep soil in a cultivated orchard. Status of tree: abandoned

**Table 2:** Fruit characteristics of Maltese olive genotypes. Data are presented as average values of the 2-year ± standard error. The values followed by different letters are significantly different for *p* < 0.05, for each trait and year. The fruits were harvested around mid-October on average over the two years.

Sample Name	Year	FW (g)	FrM (%)	(P/P)	OCFW (%)	OCDW (%)
1Bidni	2020	0.87 ± 0.04 ^ghi^	58.34 ± 0.56 ^bcd^	2.41 ± 0.01 ^e^	10.09 ± 0.17 ^c^	24.81 ± 0.50 ^de^
	2022	0.55 ± 0.02 ^jk^	52.81 ± 0.94 ^ij^	2.11 ± 0.005 ^j^	11.38 ± 0.70 ^ef^	24.14 ± 0.48 ^f^
1Bingemma Malta	2020	1.90 ± 0.07 ^d^	61.95 ± 0.04 ^a^	3.20 ± 0.07 ^c^	10.74 ± 1.00 ^c^	28.24 ± 2.62 ^cd^
	2022	4.26 ± 0.01 ^d^	57.87 ± 0.22 ^fg^	6.27 ± 0.001 ^c^	18.14 ± 0.23 ^ab^	43.05 ± 0.23 ^b^
2Gudja	2020	0.78 ± 0.03 ^ghi^	60.50 ± 0.80 ^abc^	2.22 ± 0.01 ^f^	4.31 ± 0.09 ^e^	10.90 ± 0.22 ^f^
	2022	0.72 ± 0.02 ^j^	57.24 ± 0.39 ^g^	1.75 ± 0.01 ^m^	3.80 ± 0.23 ^ij^	8.89 ± 0.08 ^i^
3Gudja	2020	1.57 ± 0.06 ^de^	58.37 ± 0.48 ^bcd^	3.23 ± 0.01 ^c^	14.59 ± 0.18 ^b^	35.05 ± 0.43 ^bc^
	2022	2.03 ± 0.03 ^f^	57.43 ± 1.04 ^fg^	3.24 ± 0.03 ^i^	15.76 ± 0.27 ^d^	37.06 ± 0.89 ^c^
2Kappara	2020	1.42 ± 0.10 ^ef^	59.52 ± 1.53 ^abc^	1.86 ± 0.02 ^g^	6.89 ± 0.22 ^d^	20.03 ± 0.54 ^e^
	2022	1.34 ± 0.01 ^h^	54.80 ± 0.03 ^hi^	1.92 ± 0.02 ^kl^	7.82 ± 0.34 ^g^	17.30 ± 0.01 ^g^
2Mtarfa	2020	0.51 ± 0.001 ^i^	50.06 ± 0.31 ^e^	1.08 ± 0.01 ^h^	2.91 ± 0.03 ^e^	6.58 ± 0.08 ^f^
	2022	0.41 ± 0.003 ^k^	56.69 ± 0.09 ^gh^	1.29 ± 0.01 ^n^	2.52 ± 0.18 ^jk^	5.82 ± 0.01 ^j^
5Mtarfa	2020	1.06 ± 0.01 ^fgh^	46.86 ± 0.71 ^f^	2.28 ± 0.05 ^ef^	15.63 ± 0.62 ^b^	29.42 ± 1.16 ^cd^
	2022	0.92 ± 0.004 ^i^	51.91 ± 0.19 ^j^	2.00 ± 0.01 ^jk^	6.82 ± 0.20 ^h^	13.06 ± 0.05 ^h^
6Mtarfa	2020	1.18 ± 0.04 d^efg^	45.23 ± 0.19 ^f^	2.96 ± 0.01 ^d^	10.51 ± 0.001 ^c^	19.19 ± 0.002 ^e^
	2022	0.40 ± 0.01 ^k^	54.41 ± 0.08 ^i^	1.72 ± 0.01 ^m^	na	na
1Pembroke	2020	0.50 ± 0.04 ^i^	48.93 ± 0.21 ^e^	1.04 ± 0.01 ^h^	2.23 ± 0.04 ^e^	4.82 ± 0.05 ^f^
	2022	0.52 ± 0.003 ^jk^	49.55 ± 0.23 ^k^	1.03 ± 0.01 ^o^	2.52 ± 0.18 ^jk^	5.00 ± 0.02 ^j^
1Plattini	2020	5.30 ± 0.05 ^b^	61.32 ± 0.18 ^ab^	6.67 ± 0.04 ^a^	15.72 ± 0.27 ^b^	39.92 ± 1.07 ^b^
	2022	5.14 ± 0.05 ^c^	64.25 ± 0.11 ^ab^	6.80 ± 0.04 ^b^	16.55 ± 0.30 ^cd^	46.28 ± 0.14 ^a^
2Qnotta	2020	2.38 ± 0.17 ^c^	62.46 ± 0.57 ^a^	2.38 ± 0.18 ^b^	9.66 ± 1.17 ^c^	25.73 ± 3.11 ^de^
	2022	1.89 ± 0.05 ^fg^	61.49 ± 0.05 ^cd^	4.08 ± 0.02 ^g^	12.47 ± 0.23 ^e^	32.39 ± 0.04 ^d^
1Wardija	2020	6.60 ± 0.19 ^a^	58.17 ± 0.15 ^cd^	6.60 ± 0.19 ^a^	24.59 ± 0.06 ^a^	58.19 ± 0.15 ^a^
	2022	7.96 ± 0.04 ^a^	58.56 ± 0.02 ^efg^	6.97 ± 0.03 ^a^	19.00 ± 0.23 ^a^	45.85 ± 0.02 ^a^
2Wardija	2020	1.49 ± 0.06 ^def^	56.40 ± 0.28 ^d^	1.72 ± 0.03 ^g^	9.33 ± 0.07 ^c^	23.21 ± 0.02 ^de^
	2022	1.29 ± 0.03 ^h^	60.30 ± 0.05 ^de^	4.79 ± 0.02 ^e^	5.60 ± 0.26 ^h^	14.10 ± 0.02 ^h^
1Caritas	2022	5.77 ± 0.10 ^b^	58.49 ± 0.01 ^efg^	5.05 ± 0.07 ^d^	17.42 ± 0.27 ^bc^	41.97 ± 0.03 ^b^
1Haz Zebbug	2022	0.52 ± 0.01 ^jk^	48.62 ± 0.45 ^k^	1.02 ± 0.01 ^o^	4.06 ± 0.23 ^i^	7.90 ± 0.07 ^i^
3Loretu	2022	1.76 ± 0.04 ^g^	62.44 ± 0.10 ^bc^	4.33 ± 0.02 ^f^	10.82 ± 0.21 ^f^	28.81 ± 0.08 ^e^
1Lunzjata	2022	0.62 ± 0.01 ^j^	64.55 ± 0.01 ^a^	1.89 ± 0.01 ^kl^	2.19 ± 0.26 ^k^	6.16 ± 0.002 ^j^
1Mellieha	2022	2.58 ± 0.07 ^e^	54.55 ± 0.19 ^i^	3.38 ± 0.01 ^h^	17.06 ± 0.23 ^bcd^	37.55 ± 0.16 ^c^
2Pembroke	2022	0.40 ± 0.01 ^k^	59.38 ± 0.31 ^ef^	1.83 ± 0.004 ^lm^	3.28 ± 0.35 ^ijk^	8.07 ± 0.06 ^i^

FW: Fresh Weight; FrM: Fruit Moisture; P/P: Pulp/Pit ratio; OCFW: Oil Content in Fresh Weight; OCDW: Oil Content in Dry Weight; na: not analyzed as the quantity of fruit was not sufficient.

**Table 3:** Descriptive statistics of squalene and sterols (mg/kg of pulp) in 19 Maltese genotypes and two crop seasons. The mean values of the three technical replicates ± standard error are reported. The values followed by different letters are significantly different for *p* < 0.05 per each year and column.

Sample Name	Year	Squalene	Total Sterols
1Bidni	2020	315.99 ± 52.78 ^c^	406.39 ± 39.08 ^ab^
	2022	122.70 ± 16.58 ^e^	177.32 ± 20.97 ^cde^
1Bingemma Malta	2020	2557.86 ± 163.96 ^b^	441.12 ± 8.87 ^ab^
	2022	5554.26 ± 769.43 ^b^	286.84 ± 6.21 ^ab^
1Pembroke	2020	54.34 ± 1.28 ^c^	61.48 ± 1.51 ^d^
	2022	21.91 ± 2.32 ^e^	76.93 ± 4.64 ^f^
1Plattini	2020	6020.81 ± 774.80 ^a^	374.37 ± 2.89 ^abc^
	2022	9558.08 ± 1096.13 ^a^	231.74 ± 15.87 ^bc^
1Wardija	2020	5697.61 ± 407.18 ^a^	405.51 ± 185.20 ^ab^
	2022	4604.50 ± 721.08 ^bc^	287.96 ± 5.84 ^ab^
2Gudja	2020	196.69 ± 12.54 ^c^	88.75 ± 4.12 ^d^
	2022	115.78 ± 6.03 ^e^	106.54 ± 4.31 ^ef^
2Kappara	2020	959.88 ± 91.76 ^c^	550.28 ± 16.22 ^a^
	2022	333.55 ± 44.31 ^e^	217.70 ± 16.72 ^bcd^
2Mtarfa	2020	63.79 ± 5.20 ^c^	68.23 ± 7.85 ^d^
	2022	31.30 ± 0.97 ^e^	106.97 ± 7.89 ^ef^
2Qnotta	2020	83.75 ± 3.96 ^c^	226.07 ± 11.57 ^bcd^
	2022	2517.33 ± 175.66 ^cd^	281.62 ± 37.27 ^ab^
2Wardija	2020	298.11 ± 20.49 ^c^	120.94 ± 8.31 ^cd^
	2022	104.53 ± 8.56 ^e^	174.95 ± 10.96 ^cde^
3Gudja	2020	377.54 ± 9.04 ^c^	180.55 ± 5.11 ^bcd^
	2022	630.15 ± 56.69 ^de^	332.73 ± 15.15 ^a^
5Mtarfa	2020	402.59 ± 5.82 ^c^	168.38 ± 1.39 ^bcd^
	2022	127.32 ± 18.49 ^e^	140.04 ± 13.15 ^def^
6Mtarfa	2020	170.81 ± 19.79 ^c^	87.82 ± 5.35 ^d^
	2022	51.72 ± 5.75 ^e^	83.74 ± 11.73 ^f^
1Caritas	2022	5185.78 ± 731.16 ^b^	266.69 ± 22.81 ^ab^
1Haz Zebbug	2022	14.39 ± 0.68 ^e^	98.38 ± 4.62 ^ef^
3Loretu	2022	2025.60 ± 250.64 ^de^	235.40 ± 15.14 ^bc^
1Lunzjata	2022	33.03 ± 6.99 ^e^	220.73 ± 2.14 ^bcd^
1Mellieha	2022	775.28 ± 141.45 ^de^	285.95 ± 27.51 ^ab^
2Pembroke	2022	19.95 ± 1.61 ^e^	71.63 ± 6.43 ^f^

## Data Availability

Data that are not already present in this manuscript and the Supporting Information will be made available upon reasonable request to the corresponding author (M.B.).

## Supplementary Materials

The following supporting information can be downloaded at: https://www.mdpi.com/article/10.3390/plants12101988/s1, Figure S1: Linear regression among some fruit traits; Table S1: Fruit phenolic profile of the 19 Maltese genotypes investigated over two years; Table S2: Fruit fatty acid profile in 19 Maltese samples over two crop seasons; Table S3: Variation of monounsaturated (MUFA), polyunsaturated (PUFA), saturated (SFA) fatty acid and OLP index indicates the ratio of oleic acid/(linoleic + palmitic acid); Figure S2: Sample locations in the Maltese Islands and pixels from two different climatic datasets; Table S4. Climatic data; Table S5: List of genotypes useful for use as table and oil olives.
