# Supplementary material for: The Ancient Olive Trees (Olea europaea L.) of the Maltese Islands: A Rich and Unexplored Patrimony to Enhance Oliviculture
Source: Plants (Basel). 2023 May 15;12(10):1988. doi: 10.3390/plants12101988 (PMC10221224; doi:10.3390/plants12101988)
Supplement: Supplementary file 1 [file plants-12-01988-s001.zip › plants-2349468-supplementary.pdf]

The ancient olive trees (*Olea europaea L.*) of the Maltese Islands: a rich and unexplored patrimony to enhance oliviculture

Valentina Passeri [1]; Clayton Sammut [2]; David Mifsud [2]; Andrea Domesi [1]; Vitale Stanzione [1]; Luciana Baldoni [3]; Soraya Mousavi [3]; Roberto Mariotti [3]; Saverio Pandolfi [3], Nicola Cinosi[4], Franco Famiani [4]; Marina Bufacchi [1,*]

Supplementary Information

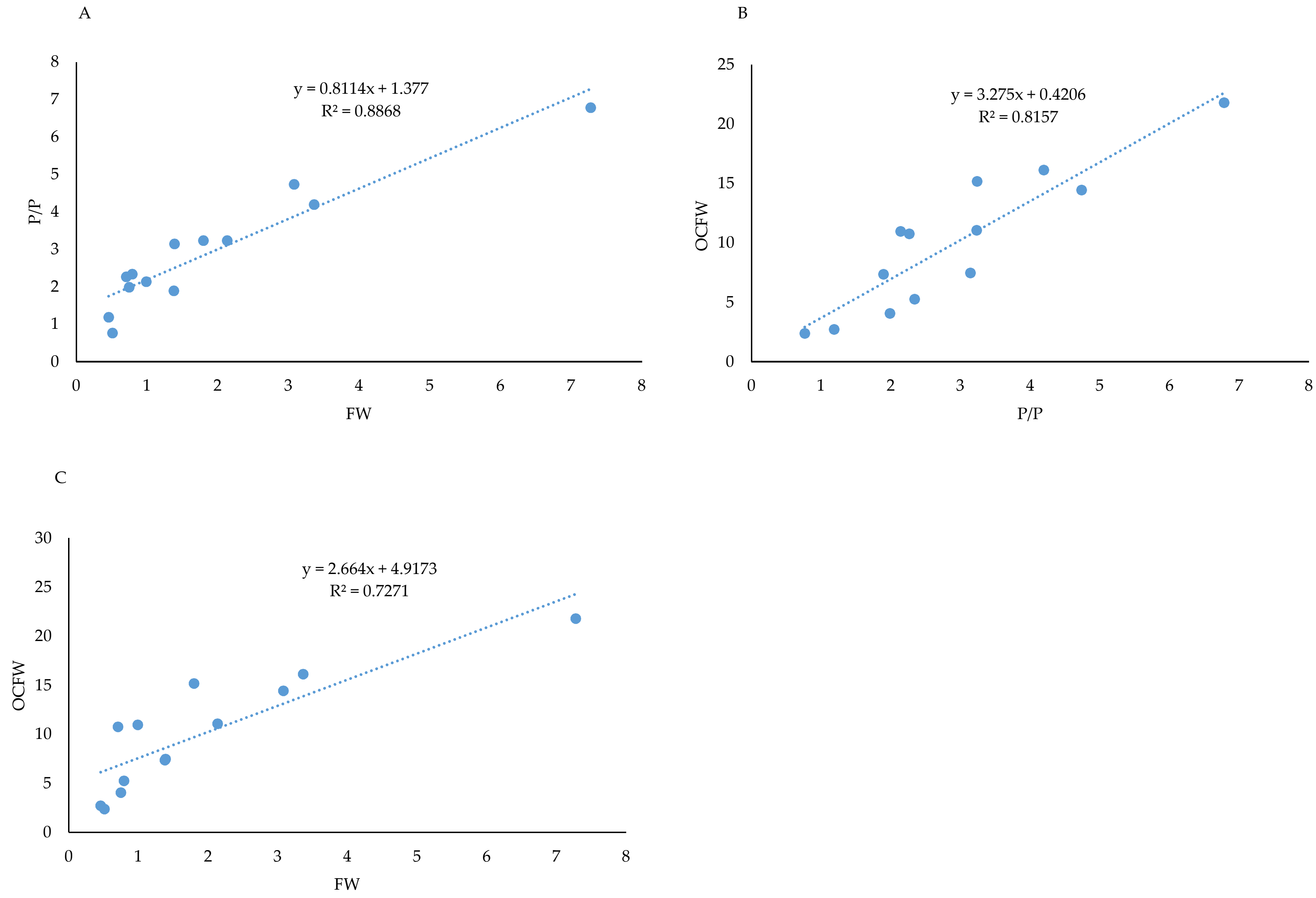

Figure S1. Linear regression among some fruit traits. A: pulp/pit ratio (P/P) and fresh Weight (FW); B: oil content in fresh Weight (OCFW) versus pulp/pit ratio (P/P); C: oil content in fresh Weight (OCFW) versus (FW). Scatter plot represents the regression between the mean values obtained from 13 genotypes over the two years.

Table S1. Fruit phenolic profile of 19 Maltese genotypes investigated over two years. Data are expressed as mg/kg of pulp and they represent the average values ± standard error. In each column, values followed by different letters are significantly different for $p < 0.05$, for each year.

Sample name	Year	HTYR	TYR	VAN_AC	CAF_AC	D_OLE	VERB	OLEAC	RUT	ISOV	LUT7G	OLEU	OLEOC	LIG	OLEU_AG	TOT_PHE
1Bidni	2020	479.3±40.0^{def}	113.4±6.6^{b}	110.1±1.0^{c}	nd	335.9±36.6^{de}	112.5±34.1^{f}	605.4±15.2^{d}	694.8±59.1^{bcd}	nd	88.0±9.0^{cd}	4889.7±1,302.1^{bc}	445.05±45.79^{abcde}	527.62±16.57^{bc}	8726.68±509.41^{hi}	17,128.45±1,863.69^{de}
	2022	597.9±74.8^{d}	78.6±11.0^{c}	108.4±9.8^{de}	nd	184.0±90.8^{b}	66.7±12.4^{g}	1452.1±114.1^{ghi}	913.1±113.1^{cd}	nd	156.2±19.1^{bcde}	8057.7±1,175.5^{def}	111.61±3.04^{d}	1067.64±200.74^{de}	30,528.08±5,144.78^{cde}	43,920.95±6,404.13^{e}
1Bingemma Malta	2020	385.4±21.2^{ef}	93.6±9.6^{b}	70.8±11.0^{c}	nd	901.8±224.9^{bcd}	1678.3±144.2^{de}	3897.2±316.5^{abcd}	400.1±27.1^{ef}	nd	34.6±11.6^{cd}	1863.7±531.9^{c}	340.38±135.27^{cde}	600.26±245.96^{bc}	11,137.08±57.12^{ghi}	21,402.99±1,195.87^{de}
	2022	136.6±11.3^{fg}	nd	73.5±1.4^{fgh}	nd	nd	297.0±88.2^{fg}	239.3±36.7^{j}	376.4±82.6^{efgh}	nd	196.0±42.4^{bc}	762.9±164.8^{ef}	216.36±49.88^{cd}	27.99±2.46^{f}	5287.58±637.42^{h}	7613.76±869.76^{gh}
1Pembroke	2020	847.7±58.0^{cd}	90.0±1.3^{b}	177.4±9.6^{b}	nd	594.8±15.4^{cde}	6257.0±567.5^{a}	900.9±33.4^{cd}	1131.6±35.1^{a}	3999.4±438.2	81.4±5.2^{cd}	13,369.6±387.99^{abc}	261.07±2.64^{cde}	4818.07±148.99^{a}	71,580.00±235.57^{a}	104,109.93±1,610.01^{a}
	2022	1033.1±61.9^{c}	111.9±1.3^{c}	225.9±4.0	nd	nd	3838.9±282.3^{b}	1440.5±64.3^{ghi}	616.8±71.4^{def}	2486.4±421.6^{a}	nd	7027.63±1,952.51^{def}	154.09±77.37^{d}	914.50±178.90^{ef}	45,434.66±2,093.17^{b}	63,917.93±4,178.93^{cd}
1Plattini	2020	195.0±26.1^{f}	nd	76.2±4.0^{c}	nd	nd	349.9±12.2^{f}	nd	125.0±11.9^{g}	nd	17.8±4.8^{d}	11,198.45±2,523.10^{abc}	765.10±50.33^{ab}	nd	6158.25±829.02^{ij}	19,433.35±3,166.51^{de}
	2022	124. 6±3.6^{g}	nd	66.3±0.5^{h}	nd	37.1±2.5^{b}	128.4±54.8^{fg}	181.6±30.7^{j}	199.2±21.8^{gh}	nd	93.5±7.4^{cde}	8348.90±1,092.87^{def}	515.15±57.35^{bc}	nd	7263.09±492.01^{h}	16,957.72±1,585.56^{fgh}
1Wardija	2020	1311.0±89.5^{b}	nd	111.7±4.3^{c}	nd	186.3±3.0^{e}	678.5±2.0^{def}	nd	884.6±57.4^{abc}	nd	675.2±69.6^{a}	4,592.23±182.50^{c}	838.14±112.40^{a}	nd	2281.69±419.26^{j}	11,559.41±269.62^{e}
	2022	1236.1±11.6^{bc}	nd	71.7±2.6^{fgh}	nd	nd	1,217.4±273.8^{ef}	nd	484.2±36.1^{efg}	nd	183.3±29.9^{bcd}	460.33±50.12^{f}	91.07±46.47^{d}	nd	2569.54±1,170.61^{h}	6313.59±984.45^{h}
2Gudja	2020	358.0±8.2^{ef}	nd	101.66±2.22^{c}	nd	1278.5±216.5^{bc}	123.9±33.5^{f}	6752.1±1133.4^{ab}	586.1±15.9^{def}	nd	47.1±4.1^{cd}	10,203.47±165.78^{abc}	412.38±67.07^{abcde}	1003.91±391.86^{bc}	22,964.34±1,373.95^{d}	43,831.46±2,974.97^{c}
	2022	314.7±24.5^{defg}	nd	94.9±2.2^{ef}	nd	nd	56.9±11.9^{g}	4177.4±227.6^{cd}	176.3±14.2^{gh}	nd	nd	2921.86±632.25^{ef}	223.94±29.09^{cd}	288.66±28.44^{f}	18,423.71±524.143^{fg}	26,678.42±1132.92^{f}
2Kappara	2020	509.8±26.0^{def}	15.0±0.3^{c}	nd	nd	17,195.7±29.1^{a}	1840.3±102.2^{cd}	3091.0±141.8^{bcd}	656.5±27.3^{cde}	nd	13.1±3.9^{d}	3356.78±68.46^{c}	132.92±37.97^{e}	65.79±4.10^{c}	2072.72±207.65^{j}	28,949.77±31.40^{cde}
	2022	304.5±95.6^{defg}	nd	266.9±7.6^{a}	nd	nd	3071.1±611.0^{bc}	1654.0±272.5^{gh}	709.7±121.8^{de}	nd	53.1±15.0^{cde}	11,580.73±1,625.67^{d}	241.87±21.37^{cd}	719.14±71.35^{ef}	42,042.38±2,255.37^{b}	60,643.34±3,597.60^{de}
2Mtarfa	2020	1,054.9±139.3^{bc}	nd	212.8±15.78^{b}	13.8±1.4^{a}	nd	99.8±5.7^{f}	4719.0±126.0^{abc}	956.8±73.2^{ab}	nd	304.7±53.2^{b}	23,954.20±9,843.46^{a}	651.97±176.63^{abc}	3894.19±313.07^{a}	56,471.10±453.18^{b}	92,333.16±9,966.60^{ab}
	2022	437.1±37.2^{defg}	nd	155.5±6.4^{c}	53.7±4.7^{a}	nd	471.5±21.8^{fg}	8762.2±177.5^{b}	1507.8±50.9^{b}	nd	414.9±22.3^{a}	30,188.51±1,452.20^{b}	881.58±198.73^{a}	3485.24±94.90^{ab}	71,512.05±2,737.40^{a}	117,870.02±3,552.94^{b}
2Qnotta	2020	605.3±39.5^{de}	nd	96.7±3.5^{c}	nd	nd	497.5±7.2^{f}	1081.8±318.0^{cd}	327.3±69.6^{fg}	nd	70.1±1.9^{cd}	4502.05±660.76^{c}	230.87±13.37^{cde}	331.82±25.77^{bc}	16,286.08±307.01^{efg}	24,029.45±733.81^{cde}
	2022	332.6±40.8^{defg}	15.4±2.0^{d}	102.103.6^{de}	nd	nd	35.5±6.5^{g}	2857.8±169.0^{ef}	271.8±22.1f^{gh}	nd	100.4±17.1^{cde}	2176.53±634.64^{ef}	74.03±11.36^{d}	419.40±38.34^{ef}	17,412.88±1,569.59^{fg}	23,798.63±2,454.36^{fg}
2Wardija	2020	684.2±29.5^{cde}	nd	114.3±14.2^{c}	nd	1074.6±208.5^{bc}	43.9±4.0^{f}	871.1±248.9^{cd}	374.4±52.1^{fg}	nd	123.5±17.6^{cd}	16,072.58±1,075.05^{abc}	172.66±37.98^{de}	467.61±64.70^{bc}	17,883.24±2,486.99^{de}	37,882.05±4,172.59^{cd}
	2022	477.9±26.3^{e}	nd	105.7±2.6^{de}	nd	2751.7±281.1^{a}	1108.6±122.2^{efg}	3722.0±324.9^{de}	728.7±57.5^{de}	nd	210.0±38.1^{bc}	8505.79±1,623.68^{de}	267.50±39.80^{cd}	410.69±13.14^{ef}	28,455.72±1,320.44^{de}	46,744.18±2,377.09^{e}
3Gudja	2020	1714.4±79.2^{a}	374.6±42.7^{a}	118.9±1.1^{c}	nd	1460.3±264.2^{b}	3019.7±343.8^{b}	2746.7±28.7^{bcd}	600.06±95.89^{def}	nd	51.7±5.1^{cd}	3097.88±608.63^{c}	157.25±14.39^{de}	319.20±80.17^{bc}	11,629.60±203.26^{fgh}	25,290.36±298.16^{cde}
	2022	1807.1±188.6^{a}	188.8±13.0^{b}	117.2±2.2^{de}	nd	96.6±51.8^{b}	2998.7±205.1^{bcd}	236.8±51.7^{j}	484.4±32.3^{efg}	nd	29.3±7.9^{de}	2088.33±995.97^{ef}	127.17±17.82^{d}	376.79±105.90^{f}	7081.91±972.41^{h}	16,199.02±1,191.78^{fgh}
5Mtarfa	2020	473.8±60.0^{ef}	nd	262.8±2.7^{a}	nd	nd	822.4±26.3^{def}	4122.97±99.8^{abcd}	487.9±55.1^{def}	nd	116.3±24.4^{cd}	7553.47±463.32^{abc}	377.83±30.63^{bcde}	1058.72±239.49^{b}	17,015.57±311.00^{ef}	32,291.64±213.49^{cde}
	2022	356.4±35.7^{defg}	16.5±5.2^{d}	227.4±4.4^{b}	nd	nd	1913.7±65.9^{de}	4767.4±272.7^{c}	981.1±55.12^{cd}	nd	252.1±68.8^{b}	31,378.96±2,709.74^{b}	393.37±25.57^{cd}	1527.16±184.51^{cde}	37,188.10±1,111.08^{bcd}	79,612.65±2,216.82^{c}
6Mtarfa	2020	1347.3u±116.4^{ab}	nd	216.8±18.1^{ab}	17.7±2.3^{a}	nd	2776.1±183.2^{bc}	7384.0±2411.8^{a}	910.3±2.0^{abc}	nd	170.8±38.5^{bc}	21,561.79±2,579.60^{ab}	579.97±71.08^{abcd}	406.52±57.358^{bc}	44,508.42±1,540.03^{c}	79,879.68±6,901.04^{b}
	2022	629.2±25.3^{d}	173.5±3.6^{b}	177.6±3.2^{c}	nd	229.3±94.1^{b}	3817.8±171.5^{b}	11,825.93332.7^{a}	618.6±46.0^{def}	nd	179.0±26.4^{bcd}	54,405.29±2,709.03^{a}	1008.93±42.28^{a}	2379.47±567.01^{bc}	67,783.23±2,372.83^{a}	143,227.81±5,466.86^{a}
1Caritas	2022	261.1±21.1^{efg}	nd	69.2±0.8^{gh}	nd	nd	138.6±47.4^{fg}	nd	721.6±125.7^{de}	nd	413.4±66.1^{a}	3257.16±801.80^{ef}	825.09±78.43^{ab}	nd	4512.15±503.02^{h}	101,98.28±1,474.44^{fgh}
1Haz Zebbug	2022	1103.6±48.6^{c}	160.4±21.4^{b}	238.7±7.5^{b}	21.5±0.6^{b}	nd	663.7±19.4^{fg}	3226.7±109.0^{def}	1101.0±93.0^{c}	nd	nd	3688.55±499.67^{def}	153.25±27.86^{d}	144.28±72.749^{f}	41,648.92±1,754.11^{b}	52,773.72±2,286.04^{de}
3Loretu	2022	394.4±50.7^{defg}	nd	94.3±4.7^{efg}	nd	nd	11.5±1.3^{g}	2270.4±151.9^{fg}	54.08±33.42^{h}	nd	8.9±5.4^{e}	519.16±80.83^{ef}	134.11±4.86^{d}	287.32±45.86^{f}	11,095.95±922.90^{gh}	14,870.08±1,278.03^{fgh}
1Lunzjata	2022	1557.1±60.1^{ab}	41.9±5.9^{d}	124.6±1.3^{d}	2.0±0.8^{c}	388.7±103.9^{b}	527.2±21.2^{fg}	632.1±186.1^{ij}	176.3±42.2^{gh}	176.2±70.2^{b}	14.9±3.2^{e}	468.50±90.43^{f}	121.77±21.56^{d}	260.17±27.02^{f}	4611.73±332.98^{h}	9103.30±829.41^{gh}
1Mellieha	2022	517.9±43.8^{de}	17.3±0.7^{d}	94.3±7.9^{efg}	22.4±3.1^{b}	nd	2171.1±287.4^{cde}	1057.0±33.1^{hij}	618.6±51.6^{def}	nd	78.1±16.1^{cde}	20,761.07±3147.92^{c}	212.57±11.33^{cd}	2094.73±229.59^{cd}	25,127.53±1,141.61^{ef}	52,772.62±4,835.80^{de}
2Pembroke	2022	457.4±22.2^{def}	334.0±7.9^{a}	118.6±2.5^{de}	24.4±2.2^{b}	nd	8043.8±312.7^{a}	2268.4±211.9^{fg}	2416.1±64.4^{a}	nd	514.4±30.0^{a}	5936.73±1,278.07^{def}	997.37±73.59^{a}	3565.94±563.53^{a}	40,010.35±1,585.04^{bc}	64,687.58±3,583.48^{cd}

For each sample, total phenols represent the sum of all fractionated phenols.
nd: not detected
OLEU_AG: oleuropein-aglycone; OLEU: oleuropein; HTYR: Hydroxytyrosol; TYR: tyrosol; VAN_AC: vanillic acid; CAF_AC: caffeic acid; D_OLE: dimethyl-oleuropein; VERB: verbascoside; OLEAC: oleacein; RUT: rutin; ISOV: isoverbascoside; LUT7G: lutein 7-glycoside; OLEOC: oleocanthal; LIG: ligstroside; TOT_PHE: total fractionated phenols

Table S2. Fruit fatty acid profile in 19 Maltese samples over 2 crop seasons. Data are presented as average values of two-years ± standard error. In each column and for each year, the values followed by different letters are significantly different for $p < 0.05$.

Sample name	Year	C16:0	C16:1	C17:1	C18:0	C18:1	C18:2	C18:3	C20:0	C20:1n9	C20:2	C20:3n6	C21:0	C20:4n6	C20:3n3
1Bidni	2020	17.70±0.10^{cd}	1.40±0.22^{cde}	1.49±0.18^{d}	2.27±0.07^{b}	60.37±0.72^{bc}	12.25±0.46^{cd}	1.97±0.45^{bc}	nd	nd	nd	nd	1.32±0.66	1.22±0.21^{de}	nd
	2022	16.47±0.15^{ef}	1.55±0.05^{cde}	0.89±0.30^{d}	2.27±0.04^{efgh}	67.35±0.75^{ab}	7.17±0.05^{ef}	1.50±0.01^{a}	0.35±0.06^{bcd}	nd	0.18±0.09^{f}	nd	1.52±0.83^{ef}	1.65±0.15^{de}	nd
1Bingemma Malta	2020	15.70±0.20^{e}	0.85±0.01^{e}	1.46±0.02^{d}	1.21±0.12^{d}	50.33±0.38^{f}	8.22±0.19^{ef}	4.45±0.17^{a}	nd	nd	nd	9.07±0.25^{a}	nd	6.86±0.17^{a}	nd
	2022	17.42±0.23^{def}	0.88±0.03^{g}	nd	2.74±0.01^{bcd}	70.48±0.36^{a}	6.31±0.15^{fg}	1.41±0.09^{hi}	0.41±0.01^{bcd}	0.34±0.06^{b}	nd	nd	nd	nd	nd
1Pembroke	2020	14.25±0.45^{f}	0.99±0.2^{de}	2.02±0.64^{cd}	1.25±0.12^{d}	45.50±1.85^{g}	7.63±0.08^{efg}	4.15±0.11^{a}	nd	nd	nd	8.87±0.47^{a}	nd	6.77±0.40^{a}	8.56±0.52^{a}
	2022	18.59±0.17^{cde}	1.61±0.03^{cde}	nd	1.77±0.04^{j}	57.70±0.07^{c}	9.49±0.13^{cdef}	5.21±0.08^{defg}	nd	nd	0.55±0.02^{def}	nd	2.88±0.08^{cdef}	2.19±0.11^{cde}	nd
1Plattini	2020	17.59±0.13^{cd}	1.77±0.06^{bcd}	0.70±0.08^{d}	5.86±0.23^{a}	49.66±0.69^{fg}	19.75±0.29^{a}	2.32±0.12^{b}	0.71±0.11^{b}	nd	nd	1.00±0.25^{e}	nd	0.63±0.15^{e}	nd
	2022	17.20±0.17^{def}	1.80±0.01^{cd}	0.89±0.30^{d}	3.16±0.09^{b}	60.51±2.32^{bc}	11.61±1.48^{bc}	1.96±0.27^{c}	0.47±0.02^{bcd}	0.19±0.12^{b}	0.18±0.09^{f}	1.25±0.23^{b}	nd	0.79±0.15^{de}	nd
1Wardija	2020	16.12±0.23^{e}	0.58±0.29^{e}	1.52±0.02^{d}	1.38±0.12^{cd}	52.07±0.51^{ef}	8.33±0.20^{ef}	4.45±0.17^{a}	nd	nd	nd	9.15±0.26^{a}	nd	6.95±0.18^{a}	nd
	2022	22.47±1.29^{a}	1.70±0.21^{cd}	5.16±4.95^{a}	2.52±0.10^{defg}	45.44±2.05^{d}	19.92±1.16^{a}	1.61±0.09^{ghi}	0.38±0.01^{bcd}	nd	nd	nd	0.49±0.10^{f}	0.32±0.06^{e}	nd
2Gudia	2020	19.09±0.33^{b}	3.14±0.13^{a}	4.07±0.69^{bc}	1.59±0.12^{bcd}	52.58±1.57^{ef}	7.11±0.19^{efg}	2.11±0.08^{b}	nd	nd	nd	3.87±0.53^{c}	nd	3.02±0.44^{c}	3.42±0.33^{c}
	2022	19.37±0.18^{bcd}	3.33±0.12^{b}	nd	1.68±0.04^{j}	63.19±0.51^{abc}	6.43±0.23^{fg}	2.09±0.08^{fghi}	0.23±0.01^{d}	nd	1.82±0.16^{b}	nd	1.11±0.09^{ef}	0.75±0.05^{e}	nd
2Kappara	2020	18.61±0.18^{bc}	2.31±0.14^{ab}	7.51±0.84^{a}	1.68±0.04^{bcd}	53.92±0.61^{def}	11.25±0.27^{d}	2.16±0.03^{b}	nd	nd	nd	0.87±0.13^{e}	nd	0.65±0.08^{e}	1.16±0.32^{de}
	2022	18.52±0.73^{def}	1.98±0.07^{c}	1.28±0.21^{d}	1.79±0.11^{j}	48.51±1.72^{d}	7.79±0.43^{def}	2.44±0.13^{efgh}	nd	0.50±0.17^{b}	1.43±0.42^{bc}	4.55±1.85^{a}	6.10±1.61^{bc}	5.11±0.63^{b}	nd
2Mtarfa	2020	13.28±0.30^{f}	0.83±0.11^{e}	4.85±0.26^{b}	1.63±0.14^{bcd}	52.24±0.98^{ef}	6.25±0.09^{g}	3.70±0.02^{a}	nd	nd	nd	6.52±0.44^{b}	nd	4.82±0.35^{b}	5.86±0.33^{b}
	2022	15.44±0.25^{f}	1.53±0.13^{cde}	4.28±0.88^{b}	1.88±0.04^{hij}	48.80±2.76^{d}	7.91±0.17^{def}	4.00±0.13^{c}	1.37±0.45^{a}	nd	1.45±0.19^{bc}	nd	8.56±1.75^{b}	4.75±0.45^{b}	nd
2Qnotta	2020	16.55±0.11^{de}	0.82±0.14^{e}	2.17±0.28^{cd}	1.35±0.23^{cd}	66.69±0.91^{a}	8.47±0.39^{e}	2.15±0.05^{b}	nd	nd	nd	0.62±0.19^{e}	nd	0.47±0.06^{e}	0.71±0.09^{f}
	2022	16.80±0.19^{ef}	1.13±0.02^{efg}	1.42±0.22^{d}	2.46±0.01^{defg}	65.22±1.11^{abc}	8.05±0.45^{def}	2.17±0.09^{efghi}	0.24±0.12^{d}	nd	nd	nd	1.52±0.13^{ef}	0.99±0.10^{de}	nd
2Wardija	2020	17.57±0.22^{cd}	0.57±0.31^{e}	1.38±0.38^{d}	1.78±0.28^{bcd}	57.95±0.58^{cd}	6.72±0.07^{f}	2.34±0.08^{b}	2.99±1.50^{a}	nd	nd	3.27±0.60^{cd}	nd	2.49±0.45^{cd}	2.93±0.44^{c}
	2022	20.61±0.76^{ab}c	3.40±0.27^{b}	3.57±1.24^{c}	1.90±0.05^{hij}	46.02±1.05^{d}	11.37±1.11^{bc}	2.54±0.12^{defg}	0.95±0.08^{ab}	nd	0.96±0.17^{cde}	nd	4.71±0.34^{cde}	3.97±0.71^{bc}	nd
3Gudia	2020	20.51±0.13^{a}	1.4±0.1^{cde}	0.88±0.46^{d}	2.16±0.23^{bc}	56.39±0.96^{cde}	15.66±0.81^{b}	1.10±0.05^{c}	nd	nd	nd	0.63±0.02^{e}	nd	0.49±0.26^{e}	0.81±0.06^{ef}
	2022	20.55±0.15^{abc}	1.70±0.08^{cd}	0.28±0.004^{e}	2.45±0.04^{defg}	58.87±0.90^{c}	13.50±0.45^{b}	1.33±0.18^{i}	0.30±0.02^{cd}	nd	nd	nd	0.60±0.11^{f}	0.43±0.08^{e}	nd
5Mtarfa	2020	17.82±0.01^{c}	1.92±0.02^{bc}	1.86±0.12^{d}	1.93±0.04^{bcd}	58.29±0.57^{cd}	13.56±0.27^{c}	1.17±0.03^{c}	nd	nd	nd	1.35±0.12^{e}	nd	1.15±0.10^{de}	0.96±0.05d^{ef}
	2022	17.65±0.20^{def}	1.33±0.06^{defg}	nd	2.71±0.01^{bcde}	62.29±0.92^{abc}	9.57±0.09^{cde}	1.99±0.13^{ghi}	0.56±0.13^{bcd}	0.19±0.09^{b}	0.33±0.05^{ef}	nd	1.16±0.59^{ef}	1.75±0.07^{de}	nd
6Mtarfa	2020	15.53±0.05^{e}	1.01±0.002^{de}	0.93±0.01^{d}	1.95±0.01^{bcd}	65.02±0.25^{ab}	7.72±0.06^{efg}	1.68±0.04^{bc}	nd	nd	nd	2.16±0.11^{de}	nd	1.82±0.11^{cde}	2.18±0.11^{cd}
	2022	16.23±0.16^{ef}	0.91±0.01^{fg}	0.29±0.02^{e}	2.04±0.11^{ghij}	58.42±2.03^{c}	7.19±0.30^{ef}	3.07±0.33^{cde}	0.86±0.06^{abc}	0.40±0.02^{b}	1.10±0.16^{bcd}	nd	5.57±1.03^{bcd}	3.93±0.73^{bc}	nd
1Caritas	2022	18.27±0.15^{cde}	1.29±0.02^{defg}	nd	5.14±0.04^{a}	62.29±0.92^{bc}	7.90±0.20^{def}	1.67±0.17^{ghi}	nd	nd	nd	nd	2.01±0.41^{def}	1.43±0.31^{de}	nd
1Haz Zebbug	2022	17.89±0.31^{def}	1.44±0.07^{cdef}	nd	2.24±0.10^{fghi}	61.36±1.53^{bc}	7.14±0.30^{ef}	2.98±0.22^{def}	nd	nd	0.44±0.23^{de}	nd	3.84±0.73^{cdef}	2.67±0.60^{cd}	nd
3Loretu	2022	16.38±0.04^{ef}	1.13±0.02^{efg}	1.31±0.09^{d}	2.64±0.20^{cdef}	66.68±0.13^{ab}	6.75±0.71^{efg}	2.11±0.12^{efghi}	nd	nd	nd	nd	1.79±0.29^{ef}	1.20±0.18^{de}	nd
1Lunzjata	2022	21.71±0.39^{ab}	5.05±0.12^{a}	3.78±0.03^{bc}	1.81±0.11^{ij}	58.4±0.01^{c}	3.99±0.14^{g}	3.45±0.28^{cd}	nd	nd	nd	nd	nd	1.85±0.21^{de}	nd
1Mellieha	2022	18.20±0.01^{cde}	0.86±0.01^{g}	nd	2.98±0.05^{bc}	67.61±0.59^{ab}	6.51±0.33^{fg}	1.56±0.12^{hi}	0.34±0.01^{bcd}	0.22±0.04^{b}	nd	nd	0.97±0.13^{f}	0.75±0.11^{e}	nd
2Pembroke	2022	16.57±0.09^{ef}	1.78±0.04^{cd}	nd	1.23±0.06^{k}	32.49±1.06^{e}	10.41±0.09^{bcd}	7.17±0.33^{a}	nd	2.60±0.11^{a}	2.76±0.20^{a}	nd	15.21±0.68^{a}	9.33±0.43^{a}	nd

nd: not detected

C16:0: palmitic acid; C16:1: palmitoleic acid; C17:1: cis-10-heptadecenoic acid; C18:0: stearic acid; C18:1: oleic acid; C18:2: linoleic acid; C18:3: linolenic acid; C20:0: arachidic acid; C20:1n9: cis-11-eicosenoic acid; C20:2: cis-11,14-eicosadienoic acid; C20:3n6: cis-8,11,14-eicosatrinoic acid; C21:0: henicosanoic acid; C20:4n6: arachidonic acid; C20:3n3: cis-11,14,17-eicosatrinoic acid.

Table S3. Variation of monounsaturated (MUFA), polyunsaturated (PUFA) saturated (SFA) fatty acids and OLP index indicates the ratio of oleic acid/(linoleic+palmitic acids). Data are presented as average values ± standard error. In each column, values followed by different letters are significantly different for $p < 0.05$, in 2020 and 2022.

Sample name	Year	MUFA	PUFA	SFA	MUFA/PUFA	PUFA/SFA	OLP
1Bidni	2020	63.26±0.70^{c}	15.45±0.16^{de}	21.29±0.56^{bc}	4.10±0.09^{bc}	0.73±0.01^{c}	2.02±0.01^{cd}
	2022	68.90±0.76^{ab}	10.50±0.08^{efgh}	20.61±0.71^{f}	6.57±0.11^{defg}	0.51±0.02^{efghi}	2.85±0.03^{abc}
1Bingemma Malta	2020	52.64±0.40^{f}	28.60±0.77^{b}	16.92±0.13^{e}	1.84±0.05^{fg}	1.69±0.04^{b}	2.10±0.03^{c}
	2022	71.71±0.40^{a}	7.73±0.24^{h}	20.56±0.21^{f}	9.30±0.33^{a}	0.38±0.01^{i}	2.97±0.06^{a}
1Pembroke	2020	48.51±1.04^{g}	35.99±1.45^{a}	15.50±0.42^{e}	1.35±0.08^{g}	2.33±0.16^{a}	2.08±0.05^{c}
	2022	59.31±0.04^{efgh}	17.44±0.02^{bcd}	23.24±0.06^{def}	3.40±0.002^{fg}	0.75±0.003^{abcd}	2.06±0.02^{gh}
1 Plattini	2020	52.14±0.61^{f}	23.71±0.66^{c}	24.16±0.14^{a}	2.20±0.09^{f}	0.98±0.03^{c}	1.33±0.03^{g}
	2022	63.39±2.15^{bcde}	15.78±1.92^{cde}	20.82±0.23^{f}	4.18±0.66^{efgh}	0.76±0.08^{abcd}	2.12±0.20^{fgh}
1Wardija	2020	54.17±0.37^{f}	28.88±0.81^{b}	17.50±0.17^{de}	1.88±0.04^{fg}	1.65±0.04^{b}	2.13±0.03^{c}
	2022	52.30±2.76^{h}	21.84±1.30^{b}	25.86±1.46^{bcd}	2.43±0.29^{hi}	0.84±0.005^{ab}	1.07±0.02^{j}
2Gudja	2020	59.79±1.04^{de}	19.53±1.41^{b}	20.68±0.43^{b}	3.10±0.27^{e}	0.95±0.09^{c}	2.01±0.04^{cde}
	2022	66.52±0.39^{abcd}	11.09±0.44^{efgh}	22.39±0.14^{def}	6.02±0.28^{bcde}	0.50±0.02^{efghi}	2.45±0.04^{cdefg}
2Kappara	2020	63.74±0.38^{bc}	16.09±0.35^{de}	20.17±0.28^{bcd}	3.97±0.10^{bcd}	0.80±0.02^{c}	1.81±0.01^{e}
	2022	52.27±1.66^{fgh}	21.32±2.18^{bc}	26.41±1.20^{bcd}	2.52±0.36^{ghi}	0.82±0.11^{abc}	1.84±0.01^{hi}
2Mtarfa	2020	57.92±0.86^{e}	27.16±1.06^{bc}	14.91±0.20^{e}	2.14±0.11^{fg}	1.82±0.09^{b}	2.67±0.01^{a}
	2022	54.61±2.00^{fgh}	18.12±0.42^{bcd}	27.27±1.64^{bc}	3.02±0.17^{fghi}	0.67±0.03^{bcde}	2.09±0.09^{gh}
2Qnotta	2020	69.68±0.49^{a}	12.42±0.58^{e}	17.90±0.23^{d}	5.64±0.29^{a}	0.69±0.04^{c}	2.67±0.08^{a}
	2022	67.77±0.91^{abc}	11.21±0.58^{efgh}	21.02±0.37^{ef}	6.08±0.38^{bcde}	0.53±0.02^{defghi}	2.63±0.11^{abcde}
2Wardija	2020	59.91±0.05^{de}	17.75±1.63^{d}	22.34±1.61^{abc}	3.43±0.29^{cde}	0.82±0.14^{c}	2.39±0.005^{b}
	2022	53.00±1.97^{gh}	18.84±1.57^{bc}	28.17±0.56^{b}	2.87±0.35^{fghi}	0.67±0.05^{bcde}	1.45±0.12^{ij}
3 Gudja	2020	58.63±0.59^{e}	18.69±0.71^{d}	22.67±0.15^{ab}	3.15±0.15^{de}	0.83±0.04^{c}	1.56±0.07^{f}
	2022	60.85±0.98^{cdef}	15.25±0.71^{cdef}	23.90±0.28^{cdef}	4.01±0.25^{efgh}	0.64±0.02^{bcdef}	1.73±0.06^{hi}
5Mtarfa	2020	62.06±0.53^{cd}	18.19±0.52^{d}	19.74±0.03^{c}	3.42±0.12^{cde}	0.92±0.03^{c}	1.86±0.03^{de}
	2022	64.27±0.64^{bcde}	13.64±0.03^{defg}	22.09±0.61^{def}	4.71±0.05^{defg}	0.62±0.02^{bcdefg}	2.31±0.03^{efg}
6Mtarfa	2020	66.96±0.24^{ab}	15.55±0.31^{de}	17.48±0.07^{de}	4.31±0.10^{b}	0.89±0.02^{c}	2.8±0.003^{a}
	2022	60.01±2.30^{defg}	15.29±1.48^{cdef}	24.70±0.83^{cde}	4.04±0.59^{efg}h	0.62±0.04^{cdefgh}	2.50±0.11b^{cdef}
1Caritas	2022	63.58±0.94^{bcde}	11.00±0.67^{efgh}	25.42±0.27^{bcd}	5.83±0.43^{bcde}	0.43±0.02^{fghi}	2.38±0.04^{edfg}
1Haz Zebbug	2022	62.80±1.60^{bcde}	13.24±1.29^{defg}	23.97±0.36^{cdef}	4.87±0.66^{cde}	0.55±0.05^{defghi}	2.45±0.06^{cdefg}
3Loretu	2022	69.12±0.20^{ab}	10.07±0.64^{fgh}	20.82±0.44^{f}	6.93±0.49^{bc}	0.49±0.04^{efghi}	2.89±0.10^{ab}
1Lunzjata	2022	67.21±0.14^{abc}	9.28±0.63^{gh}	23.51±0.49^{cdef}	7.31±0.55^{ab}	0.40±0.03^{ghi}	2.27±0.02^{fg}
1Mellieha	2022	68.70±0.63^{ab}	8.82±0.46^{gh}	22.48±0.16^{def}	7.84±0.51^{ab}	0.39±0.02^{hi}	2.74±0.06^{abcd}
2Pembroke	2022	36.87±0.99^{i}	29.67±0.66^{a}	33.01±0.72^{a}	1.25±0.06^{i}	0.90±0.01^{a}	1.20±0.04^{j}

A.

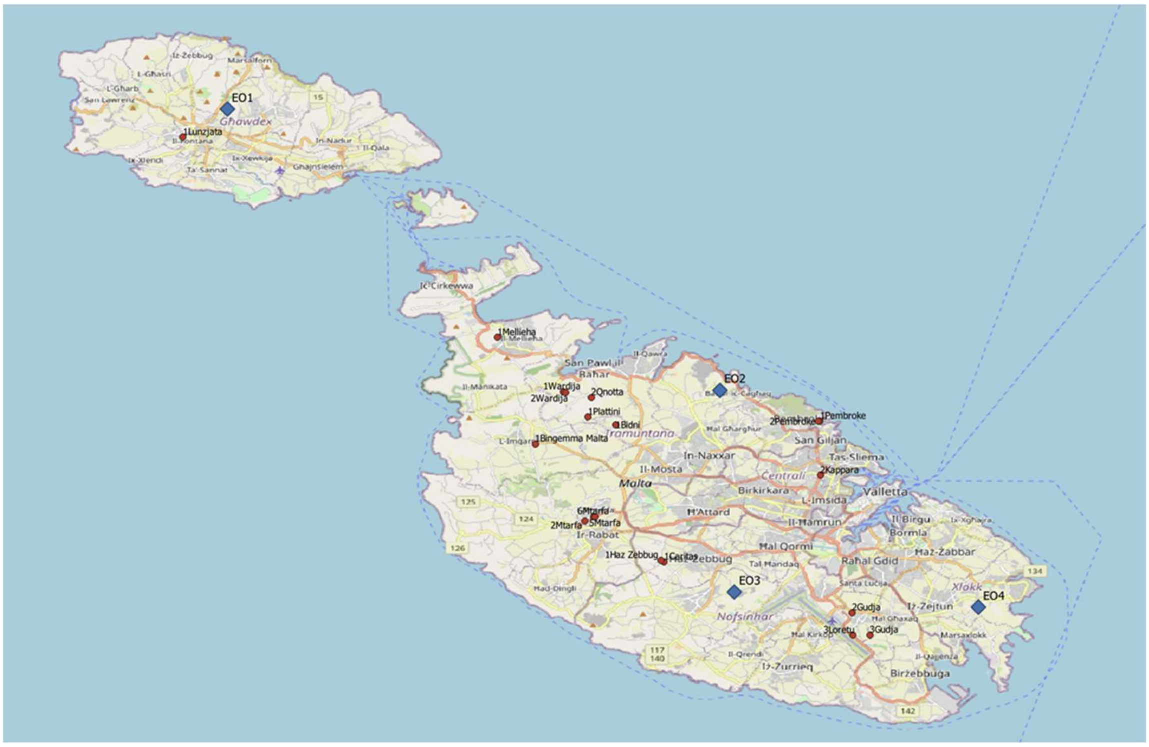

B.

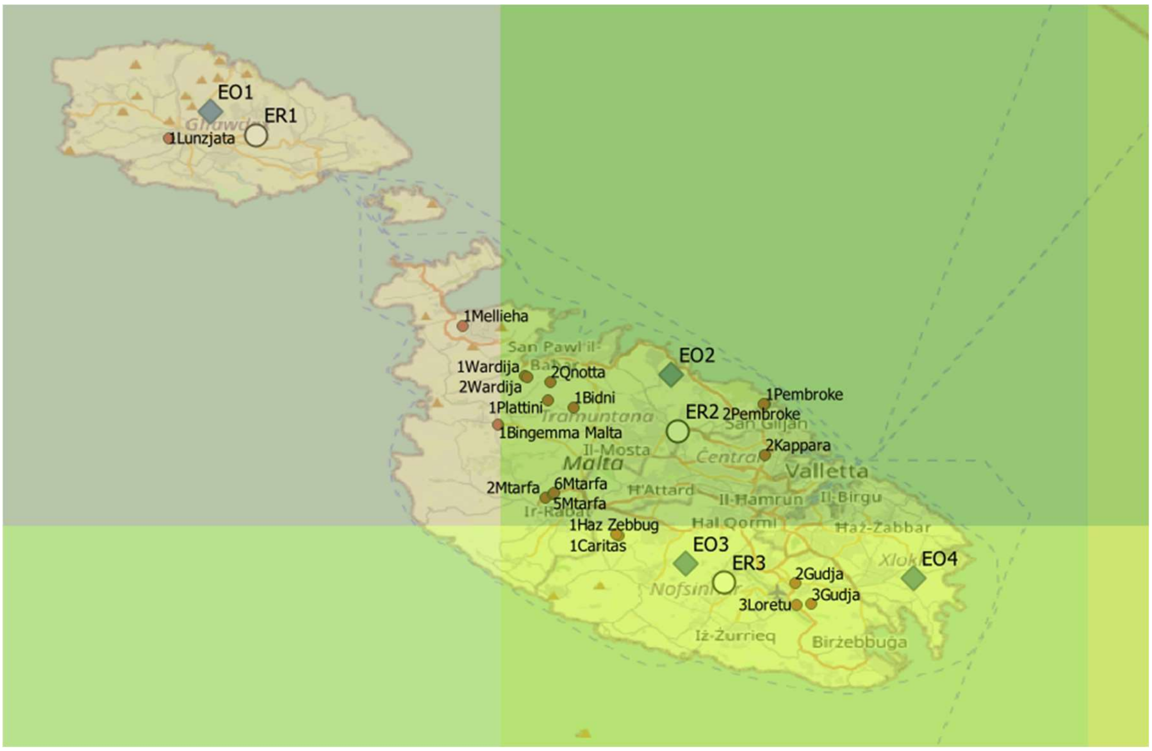

Figure S2. Samples location in the Maltese Islands and Pixels from two different climatic dataset. A.: Pixel from E-OBS (https://surfobs.climate.copernicus.eu/dataaccess/access_eobs.php). B: Pixel from ERA5 (https://cds.climate.copernicus.eu/cdsapp#!/dataset/reanalysis-era5-land?tab=overview).

Table S4. Climatic data. In A. Climatic data from the dataset E-OBS derived from interpolation of in situ observations and in B. Climatic data from the dataset ERA5 Land derived from ECMWF ERA5 climate reanalysis**

A.

Location	Year	Prec (mm)	Tmin (°C)	Tmax (°C)
EO1	2020	468	4.9	36.4
EO1	2021	769	4.2	38.2
EO2	2020	476	4.9	36.3
EO2	2021	794	4.1	38.7
EO3*	2020	479	4.8	36.1
EO3*	2021	814	4.2	38.7
EO4*	2020	474	4.8	36.1
EO4*	2021	802	4.3	38.9

*Denotes E-OBS cells without complete data series, hence the climatic indices estimates should be considered as indicative.

B.

Location	Year	Prec (mm)	Tmin (°C)	Tmax (°C)
ER1	2020	308	11.9	28.3
ER1	2021	489	9.8	29.4
ER1	2022	302	10	29.5
ER2	2020	310	11.8	28.4
ER2	2021	478	9.8	29.3
ER2	2022	290	9.8	29.4
ER3	2020	323	12	28.3
ER3	2021	481	10	29.5
ER3	2022	280	9.8	29.6

**These data being derived from model reanalysis cannot fully capture the variability of local meteorological observations and should be considered as indicative.

Prec : average annual rainfall
Tmin: average value of the annual minimum temperatures
Tmax: average value of the annual maximum temperatures

Table S5. List of genotypes useful for use as table and oil olives. The selection among the genotypes was done by evaluating the main fruit traits (pulp/pit ratio and fresh fruit weight), the oil content and the value of the oleic acid content.

	Proposed use	
**Sample genotypes**	**Table olives**	**Oil olives**
1Bidni	x	x
1Bingemma Malta	x	
1Plattini	x	
1Wardija	x	
2Kappara	x	
2Qnotta	x	x
2Wardija	x	x
3Gudja		x
1Caritas	x	
3Loretu	x	x
1Mellieha		x
